# Association between patient survival following reoperation after total hip replacement and the reason for reoperation: an analysis of 9,926 patients in the Swedish Hip Arthroplasty Register

**DOI:** 10.1080/17453674.2019.1597062

**Published:** 2019-04-01

**Authors:** Peter Cnudde, Erik Bülow, Szilard Nemes, Yosef Tyson, Maziar Mohaddes, Ola Rolfson

**Affiliations:** a Swedish Hip Arthroplasty Register, Gothenburg, Sweden;; b Department of Orthopaedics, Institute of Clinical Sciences, Sahlgrenska Academy, University of Gothenburg, Sweden;; c Department of Orthopaedics, Hywel Dda University Healthboard, Prince Philip Hospital, Llanelli, UK;; d Section of Orthopaedic Surgery, Department of Surgical Sciences, Uppsala University Hospital, Uppsala, Sweden

## Abstract

Background and purpose — The association between long-term patient survival and elective primary total hip replacement (THR) has been described extensively. The long-term survival following reoperation of THR is less well understood. We investigated the relative survival of patients undergoing reoperation following elective THR and explored an association between the indication for the reoperation and relative survival.

Patients and methods — In this observational cohort study we selected the patients who received an elective primary THR and subsequent reoperations during 1999–2017 as recorded in the Swedish Hip Arthroplasty Register. The selected cohort was followed until the end of the study period, censoring or death. The indications for 1st- and eventual 2nd-time reoperations were analyzed and the relative survival ratio of the observed survival and the expected survival was determined.

Results — There were 9,926 1st-time reoperations and of these 2,558 underwent further reoperations. At 5 years after the latest reoperation, relative survival following 1st-time reoperations was 0.94% (95% CI 0.93–0.96) and 0.90% (CI 0.87–0.92) following 2nd-time reoperations. At 5 years patients with a 1st-time reoperation for aseptic loosening had higher survival than expected; however, reoperations performed for periprosthetic fracture, dislocation, and infection had lower survival.

Interpretation — The relative survival following 1st- and 2nd-time reoperations in elective THR patients differs by reason for reoperation. The impact of reoperation on life expectancy is more obvious for infection/dislocation and periprosthetic fracture.

While the risk of dying and life expectancy following a primary THR has been studied extensively, patient survival after further surgical interventions is virtually unexplored (Jones et al. 2018, Yao et al. 2018). What happens to life expectancy if patients undergo reoperation and does the clinical indication for the reoperation influence life expectancy? So far, little is known about death following reoperation or revision after THR. The increasing age at the time of reoperation, the increased complexity of the surgery, and the timing of surgery might influence life expectancy. The relative survival method has been developed to provide better insights into the relation between a study population and a general population (Stare et al. 2005).

The Swedish Hip Arthroplasty Register (SHAR), a reliable source of information on longitudinal outcome (Kärrholm 2010, Cnudde et al. 2016) combined with national aggregated data from Statistics Sweden provide a platform for this study, which investigates the relative survival of patients undergoing reoperation following elective THR and the influence of the indication for the reoperation on the relative survival. Additionally, it investigates time- and indication-dependent patterns for 1st- and 2nd-time reoperations.

## Patients and methods

### Data sources

For this study, we used prospectively collected data on all patients who underwent a 1st-time reoperation following elective THR in 1999–2017 as recorded in SHAR. Patients who had their primary THR before 1999 were excluded. All surgical- and patient-related variables could be accessed and analysed from the database. However, for this analysis we concentrated solely on age, sex, and indication for surgery at the time of primary THR and age at the time of and indications for further operations. A reoperation is defined as any further surgery to the hip regardless of whether implant components are exchanged, removed, added or not, whereas a revision is defined as a reoperation where implant components are exchanged, removed, and/or added. Cause for reoperation was categorized into aseptic loosening, dislocation, periprosthetic fracture, infection, and other causes. Closed reductions, aspirations and isolated tissue biopsies are not included in this definition.

The selected cohort was followed until the end of the study period (December 31, 2017), censoring, or death.

### Statistics

Continuous variables were summarized as means (SD), categorical variables as percentages. Subsequently, we summarized and illustrated survival with the help of relative survival curves with 95% confidence intervals (CI) (Pohar Perme et al. 2012). We used R version 3.5 for statistical analyses (R Core Team (2018), R: A language and environment for statistical computing, Vienna, Austria, https://www.R-project.org) with the “relsurv” package for statistical analysis and applied the Pohar Perme method for calculating the relative survival.

### Relative survival

The relative survival was based on comparison of patients who underwent reoperation with aggregated data at national level from the general population (http://www.mortality.org). For any given time point onward, we estimated the relative survival ratio based on the observed survival in the patient group divided by the observed survival in general population matched on age, sex, and year of birth. The observed survival of the general population was extracted from publicly available mortality tables tabulated for birth year and sex. The formula has previously been published (Cnudde et al. 2018a). A relative survival of 1 indicates that the exposure of interest, here the reoperation or the condition causing it, does not affect the survival in any measurable way. It does not mean that all patients survive. A relative survival of less than 1 indicates excess hazard for the patients, while values above 1 indicate better survival than expected.

### Ethics, funding, and potential conflicts of interests

This study is part of a research project with the overall aim to perform a multidimensional longitudinal outcomes assessment following total hip replacement. Ethical review approval was obtained on April 7, 2014 from the Regional Ethical Review Board in Gothenburg, Sweden (entry number 271-14). The study was in part financed by grants from the Swedish state under the agreement between the Swedish government and the county councils, the ALF agreement (ALFGBG-522591). No competing interests declared. 

## Results

Using the SHAR databases, 278,309 primary THRs were identified in the study period from January 1, 1999 to December 31, 2017. There were 9,926 1st-time reoperations, of which 7,581 were 1st-time revision procedures. Of these 2,558 underwent further reoperations, of which 1,541 were subsequent revisions. There were patients undergoing subsequent procedures (up to 19 recorded reoperations and 8 revisions). Patients’ demographics and indications for reoperations are presented in Table 1. Patients who underwent reoperation for infection and aseptic loosening were generally younger at the time of their surgery than patients undergoing the procedure for dislocation and periprosthetic fracture. The indications for reoperations varied according to sex, with more females undergoing reoperations for periprosthetic fractures and dislocations whereas more males had reoperations for infection. The median follow-up time was 8.4 years (0–18) from primary surgery.

**Table 1.** Demographics of the study population. Values are mean years (SD) unless otherwise stated

	Aseptic loosening	Dislocation	Periprosthetic fracture	Infection	Other	Unknown
n	3,558	1,782	1,574	2,065	877	60
Age at primary THR	61 (11)	69 (11)	70 (11)	68 (12)	60 (13)	60 (16)
Time to 1st reoperation	8.0 (4.4)	3.2 (4.0)	5.2 (4.4)	1.6 (3.1)	4.1 (4.0)	6.4 (5.3)
Time from 1st to 2nd reoperation	2.0 (2.6)	1.7 (2.9)	1.6 (2.4)	0.6 (1.5)	1.5 (2.2)	1.4 (3.1)
Age at 1st reoperation	70 (11)	73 (11)	76 (12)	70 (11)	65 (12)	67 (16)
Age at 2nd reoperation	69 (11)	73 (11)	73 (13)	69 (11)	67 (13)	69 (14)
Women, n (%)	1,790 (50)	1,045 (59)	884 (56)	869 (42)	478 (55)	40 (57)
Dead, n (%)	566 (16)	818 (46)	711 (45)	645 (31)	136 (16)	10 (14)

p-value < 0.001 for all comparisons.

Patients undergoing 1st-time reoperation had a lower survival rate compared with the general population for the whole study period. The relative survival was 98% (CI 98–99) at 1 year, 94% (CI 93–96) at 5 years, 80% (CI 75–86) at 10 years, and 61% (CI 50–74) at 15 years. Relative survival was worse for 2nd-time reoperations compared with 1st-time reoperations with 97% (CI 96–98) at 1 year, 90% (CI 87–92) at 5 years, 69% (CI 56–85) at 10 years, and 49% (CI 34–71) at 15 years.

**Figure 1.**
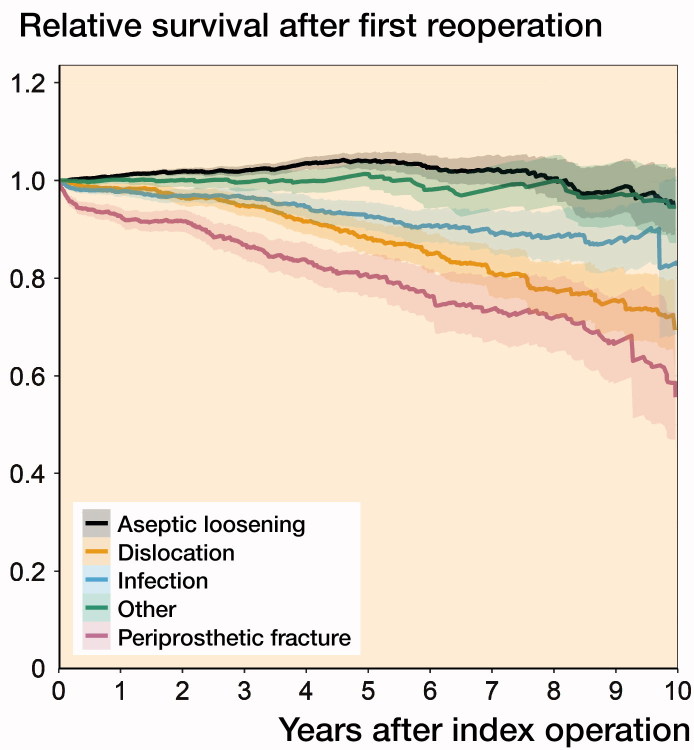
Relative survival after 1st-time reoperation per indication at the time of the reoperation (truncated at 10 years).

We stratified the relative survival per indication for reoperation (Figure 1). 1st-time reoperations for aseptic loosening had similar survival to the general population. In fact, the relative survival ratio implied a 4% increased survival compared with the general population at 5 years. Relative survival following reoperations performed for periprosthetic fracture was worse compared with aseptic loosening or other causes up to 15 years. Up to 5 years, relative survival following 1st-time reoperations for dislocation and infection was worse than for aseptic loosening and other causes (Table 2, see Supplementary data).

**Figure 2.**
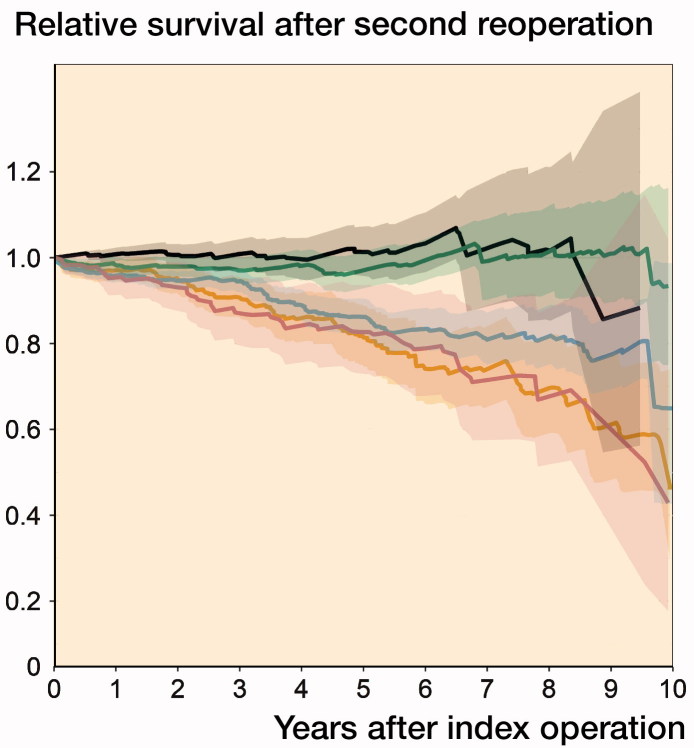
Relative survival (and confidence intervals) after the 2nd-time reoperation per indication at the time of the reoperation (truncated at 10 years).

The relative survival following a 2nd-time reoperation (Figure 2) was only marginally better if the reoperation was performed for aseptic loosening within the 1st year (Table 3, see Supplementary data). Further reoperations for infection, periprosthetic fracture, and dislocation had lower survival with the lowest survival in cases of re-reoperation for dislocation and periprosthetic fracture. The number of patients at risk is below 100 at 10 years in the group of the re-reoperations with the exception of infections and other (non-further specified) indications.

**Figure 3.**
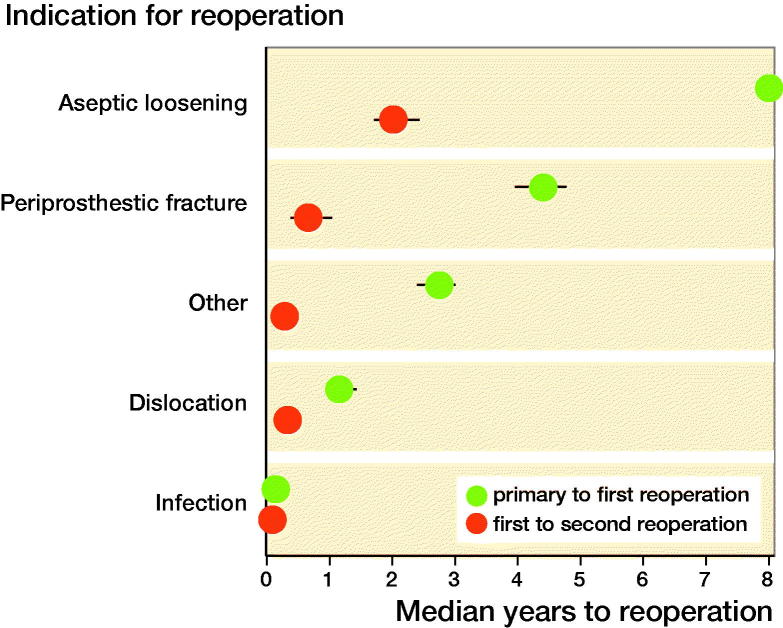
Median time between the primary THR and the 1st-time reoperation and between 1st- and 2nd-time reoperation for the different indications.

There was a difference in time between primary THR and reoperation depending on the indication for the surgery (at the time of the reoperation), with a shorter time interval in the case of infection, dislocation, and periprosthetic fracture in the 1st postoperative year. A later and a more gradual increase in cases of loosening could be seen at later stages postoperatively. Further reoperations peaked very early after the 1st re-intervention with the shortest interval when the 1st reoperation was performed for infection (Figure 3).

We also studied the influence of the clinical diagnosis at the time of primary THR on further reoperations (Table 4, see Supplementary data). Aseptic loosening was the most frequent indication at the time of reoperation when the primary THR was performed for almost all clinical indications except when a THR was performed as a result of trauma complications. Infections and dislocations frequently led to further infections for the same reasons (Table 5, see Supplementary data). Many treatment-resistant infections end up leading to an excision arthroplasty of the THR (classified as other procedure within the Tables and Figures). 

## Discussion

We found that the survival following 1st- and 2nd-time repeat surgery in elective THR is influenced by the reason for the reoperation. Patients undergoing reoperations for aseptic loosening had a survival that is better compared with the survival of the general population up to 5 years following the reoperation. Reoperations for dislocation and periprosthetic fractures were associated with a worse survival compared with the survival of the general population, also visible in the case of a further reoperation.

THR can fail for a variety of reasons in isolation or through a combination of factors. It is well known that the patient-reported outcomes after revision are worse compared with those of a primary procedure (Lubbeke et al. 2007, Postler et al. 2017). It is also likely that activity levels, as well as a potential to return to work, is affected by the revision THR (Scott et al. 2018). Nevertheless, the crude indicators of success have been re-revision/reoperation and/or mortality following the surgical procedure. The risk of further reoperations after a reoperation has been alluded to in the annual reports of the SHAR (https://shpr.registercentrum.se) and a 20% risk of a subsequent reoperation has been described. In the majority of cases a 1st reoperation occurs early within the 1st couple of years with a shortening of time interval when further reoperations are needed.

Mortality can be calculated in different ways and the effect of the procedure on life expectancy will be influenced by a multitude of factors. Short-term mortality has been described by Jones et al. (2018) based on a comparison of patients who underwent revision surgery and were compared with patients awaiting revision surgery. They found a higher mortality rate in patients who underwent revision surgery. This is likely attributed to the effect of the surgery. Yao et al. (2018) described long-term mortality following revision THR using the Mayo database and found a slightly higher mortality than in the general population. Our findings differ from the Mayo findings as we have an improved relative survival until 5 years postoperatively and from 5 years onward a survival that does not differ from the general population. The survival in patients reoperated for infections, periprosthetic fractures, and dislocations are, however, all worse than in the general population. The Mayo group combined aseptic loosening, bearing wear, and dislocation in a single group. Whilst we were not able to look at the influence of comorbidity, we did study the effect of further reoperations.

Relative survival methods have been used to describe the cancer prognosis at population level. The overall relative survival 5 years after a 1st-time reoperation is comparable with the 5-year relative survival of melanomas (94% and 95%) in females in Australia and Sweden. Our 5-year relative survival after 2nd-time reoperation is slightly worse compared with the Australian and Swedish 5-year relative survival after diagnosis of breast cancer in women (91% and 92%). The overall 10-year relative survival for all cancers in Australia and Sweden is 61% and 69%. Our 10-year relative survival after reoperation is worse than the 10-year relative survival for breast cancer in Australia and Sweden (85% and 86%) and comparable to the relative survival after cervical cancer (70% and 76%) (https://ncci.canceraustralia.gov.au/outcomes/relative-survival-rate/10-year-relative-survival and https://www.cancerfonden.se/cancer-i-siffror). These comparisons illustrate the severity of suffering THR complications leading to reoperation.

Despite the ongoing improvement in outcomes of THR, some issues remain. There is a gradual increase in patients undergoing reoperations for infection and dislocation (Cnudde et al. 2018b). These predominantly occur in the early postoperative stages and are associated with an increased mortality. There is also an accepted knowledge that revision THRs do worse than primary THRs (Espehaug et al. 1998, Lie et al. 2004). Therefore the right implant choice at the time of the primary procedure (Thien et al. 2014), management strategies at the time of the primary surgery, and subsequent surgeries will have to be developed and followed in an attempt to get the best outcomes and to avoid complications such as dislocation, infection, and periprosthetic fractures. Such complications not only put a burden on the patient but also have an impact on the scarce health care resources, and the question will have to be asked which departments and surgeons will be best placed to organise and provide the treatment and ensure the best possible outcomes.

The risk of subsequent re-reoperations is quite prominent in the case of infection and dislocation, with further surgery for the same reasons being the most common indication if surgery takes place. This reiterates the complexity of this problem and the need to develop management pathways for the prevention and adequate treatment of infections and dislocations. Bigger head size and dual mobility cups have been advocated in the case of dislocation (Mohaddes et al. 2017). New pathways advocating a timely diagnosis, rigorous debridement, local delivery of antibiotics, and prolonged use of systemic antibiotics are now considered the mainstay of treatment protocols in the case of periprosthetic joint infections.

### Strengths and limitations

This study is based on data from a validated national quality register with linkage to the administrative databases.We deliberately selected the period 1999–2017 as 1999 coincides with the year when more details of the implants used became available within the SHAR, and represents the time when increased use of more contemporary implants and head sizes was noticed, reflecting current practice. We acknowledge that selecting only reoperations following primary surgeries performed during this period does not reflect the actual proportion of different reasons for reoperations undertaken in Sweden; the distribution is skewed towards early and mid-term complications.

In our analysis there was no matching for comorbidity and socioeconomic status. A previous study of our research group has described the effect of both comorbidity and socioeconomic status on both mortality and revision after THR (Cnudde et al. 2018c). We are aware that the indication at the time of primary surgery probably influences the reason for subsequent revisions with those who have a THR for complications after trauma or acute fracture having a higher risk of dislocation, periprosthetic fracture, and infection (Cnudde et al. 2018c).

Whether the risk of earlier death is linked to the reoperation per se or to the increased risk of reoperation in frail patients is something that will need further study.

In summary the impact of reoperation on life expectancy is obvious for infection/dislocation and periprosthetic fracture. Treatment strategies should be developed to prevent these complications occurring and surgical pathways for the reoperations should be developed in order to improve outcomes for patients.

### Supplementary data

Tables 2–5 are available as supplementary data in the online version of this article, http://dx.doi.org/10.1080/17453674.2019.1597062

PC, SN, and OR conceived and planned the study. EB and SN processed the data and performed the statistical analysis. PC prepared the 1st draft of the manuscript. All authors cooperated in the analysis and interpretation of the data and the writing of the manuscript.

The authors would like to thank the orthopedic teams at the different centres in Sweden as well as the register coordinators at the Registercentrum Västra Götaland for their ongoing support with the data provision and collection.

*Acta* thanks Ove Furnes and Per Kjaersgaard-Andersenfor help with peer review of this study.

## Supplementary Material

Supplemental Material

## Supplementary data

Tables 2–5 are available as supplementary data in the online version of this article, http://dx.doi.org/10.1080/17453674.2019.1597062

## References

[CIT0001] Cnudde P, Rolfson O, Nemes S, Karrholm J, Rehnberg C, Rogmark C, Timperley J, Garellick G Linking Swedish health data registers to establish a research database and a shared decision-making tool in hip replacement. BMC Musculoskelet Disord 2016; 17(1): 414. 27716136 10.1186/s12891-016-1262-x PMC5050595

[CIT0002] Cnudde P, Rolfson O, Timperley A J, Garland A, Karrholm J, Garellick G, Nemes S Do patients live longer after THA and is the relative survival diagnosis-specific? Clin Orthop Relat Res2018a; 476(6): 1166–75. 29489471 10.1007/s11999.0000000000000097 PMC6263594

[CIT0003] Cnudde P, Nemes S, Bulow E, Timperley J, Malchau H, Karrholm J, Garellick G, Rolfson O Trends in hip replacements between 1999 and 2012 in Sweden. J Orthop Res2018b; 36(1): 432–42.1 28845900 10.1002/jor.23711 PMC5873269

[CIT0004] Cnudde P H J, Nemes S, Bulow E, Timperley A J, Whitehouse S L, Karrholm J, Rolfson O Risk of further surgery on the same or opposite side and mortality after primary total hip arthroplasty: a multi-state analysis of 133,654 patients from the Swedish Hip Arthroplasty Register. Acta Orthop2018c; 89(4): 386–93. 29792086 10.1080/17453674.2018.1475179 PMC6066773

[CIT0005] Espehaug B, Havelin L I, Engesaeter L B, Langeland N, Vollset S E Patient satisfaction and function after primary and revision total hip replacement. Clin Orthop Relat Res 1998; (351): 135–48. 9646756

[CIT0006] Jones M D, Parry M, Whitehouse M R, Blom A W Early death following revision total hip arthroplasty. Hip Int 2018; 28(4): 400–6. 29218686 10.5301/hipint.5000593

[CIT0007] Kärrholm J The Swedish Hip Arthroplasty Register (http://www.shpr.se). Acta Orthop 2010; 81(1): 3–4. 20170435 10.3109/17453671003635918 PMC2856196

[CIT0008] Lie S A, Havelin L I, Furnes O N, Engesaeter L B, Vollset S E Failure rates for 4762 revision total hip arthroplasties in the Norwegian Arthroplasty Register. J Bone Joint Surg Br 2004; 86(4): 504–9. 15174543

[CIT0009] Lubbeke A, Katz J N, Perneger T V, Hoffmeyer P Primary and revision hip arthroplasty: 5-year outcomes and influence of age and comorbidity. J Rheumatol 2007; 34(2): 394–400. 17143967

[CIT0010] Mohaddes M, Cnudde P, Rolfson O, Wall A, Karrholm J Use of dual-mobility cup in revision hip arthroplasty reduces the risk for further dislocation: analysis of seven hundred and ninety one first-time revisions performed due to dislocation, reported to the Swedish Hip Arthroplasty Register. Int Orthop 2017; 41(3): 583–8. 28078362 10.1007/s00264-016-3381-2

[CIT0011] Pohar Perme M, Stare J, Esteve J On estimation in relative survival. Biometrics 2012; 68(1): 113–20. 21689081 10.1111/j.1541-0420.2011.01640.x

[CIT0012] Postler A E, Beyer F, Wegner T, Lutzner J, Hartmann A, Ojodu I, Gunther K P. Patient-reported outcomes after revision surgery compared to primary total hip arthroplasty. Hip Int 2017; 27(2): 180–6. 27886353 10.5301/hipint.5000436

[CIT0013] Scott C E H, Turnbull G S, Powell-Bowns M F R, MacDonald D J, Breusch S J Activity levels and return to work after revision total hip and knee arthroplasty in patients under 65 years of age. Bone Joint J 2018; 100-B(8): 1043–53. 30062939 10.1302/0301-620X.100B8.BJJ-2017-1557.R2

[CIT0014] Stare J, Henderson R, Pohar M An individual measure of relative survival. J Roy Stat Soc Series C (Applied Statistics) 2005; 54(1): 115–26.

[CIT0015] Thien T M, Chatziagorou G, Garellick G, Furnes O, Havelin L I, Makela K, Overgaard S, Pedersen A, Eskelinen A, Pulkkinen P, Karrholm J Periprosthetic femoral fracture within two years after total hip replacement: analysis of 437,629 operations in the Nordic Arthroplasty Register Association database. J Bone Joint Surg Am 2014; 96(19): e167. 25274795 10.2106/JBJS.M.00643

[CIT0016] Yao J J, Maradit Kremers H, Abdel M P, Larson D R, Ransom J E, Berry D J, Lewallen D G Long-term mortality after revision THA. Clin Orthop Relat Res 2018; 476(2): 420–6. 29389795 10.1007/s11999.0000000000000030 PMC6259686

