# Supplementary material for: Association between patient survival following reoperation after total hip replacement and the reason for reoperation: an analysis of 9,926 patients in the Swedish Hip Arthroplasty Register
Source: Acta Orthop. 2019 Apr 1;90(3):226–30. doi: 10.1080/17453674.2019.1597062 (PMC6534231; doi:10.1080/17453674.2019.1597062)
Supplement: Supplemental Material [file IORT_A_1597062_SM5968.pdf]

Supplementary data

Table 2. Relative survival rates (and confidence intervals) for different indications for 1st-time reoperation at the time of surgery at different follow-up times

	Relative survival rate (CI) at<br>1 year	 5 years	 10 years	 15 years
Aseptic loosening	1.01 (1.01–1.01)	1.04 (1.02–1.06)	0.96 (0.89–1.03)	0.82 (0.64–1.06)
Dislocation	0.98 (0.97–0.99)	0.88 (0.85–0.91)	0.69 (0.61–0.79)	0.53 (0.37–0.75)
Periprosthetic fracture	0.93 (0.91–0.94)	0.80 (0.76–0.85)	0.56 (0.44–0.71)	0.38 (0.24–0.61)
Infection	0.98 (0.97–0.99)	0.93 (0.90–0.96)	0.83 (0.68–1.00)	0.56 (0.37–0.84)
Other	1.00 (0.99–1.01)	1.01 (0.99–1.04)	0.95 (0.87–1.03)	0.88 (0.70–1.09)

Table 3. Relative survival (and confidence intervals) for different indications at 2nd-time reoperation at different follow-up times

	Relative survival rate (CI) at<br>1 year	 5 years	 10 years	 15 years
Aseptic loosening	1.01 (1.01–1.02)	1.01 (0.96–1.07)	0.92 (0.59–1.45)	0.81 (0.42–1.56)
Dislocation	0.97 (0.95–1.00)	0.81 (0.75–0.89)	0.46 (0.29–0.73)	0.22 (0.11–0.43)
Periprosthetic fracture	0.95 (0.91–0.99)	0.83 (0.73–0.94)	0.43 (0.18–1.04)	0.41 (0.16–1.03)
Infection	0.96 (0.94–0.98)	0.86 (0.82–0.91)	0.65 (0.43–0.99)	0.73 (0.46–1.15)
Other	0.98 (0.97–1.00)	0.97 (0.93–1.01)	0.94 (0.75–1.17)	0.43 (0.19–0.99)

Table 4. Reasons for 1st-time reoperation versus the original diagnosis that precipitated the THR

Original diagnosis	Reasons for 1st-time reoperation<br>Aseptic loosening	Dislocation	Periprosthetic fracture	Infection	Other	Unknown
Primary osteoarthritis	2,904	1,306	1,111	1,584	693	53
Childhood hip disorder	116	44	29	48	46	4
Complication after trauma	154	257	243	227	52	4
Femoral head necrosis	115	68	60	66	17	1
Inflammatory joint disease	120	48	54	63	20	4
Secondary osteoarthritis	149	59	77	77	49	4

Table 5. Reasons for 2nd-time reoperations versus the reason for the 1st-time reoperation

Reasons for 1st-time reoperation	Reasons for 2nd-time reoperation<br>Aseptic loosening	Dislocation	Periprosthetic fracture	Infection	Other	None
Aseptic loosening	191	89	70	127	66	3,015
Dislocation	42	286	30	110	21	1,293
Periprosthetic fracture	44	27	64	51	77	1,311
Infection	29	16	22	633	342	1,023
Other	40	22	15	72	60	668
Unknown	1	0	1	1	0	67
